# *Efr3a* Insufficiency Attenuates the Degeneration of Spiral Ganglion Neurons after Hair Cell Loss

**DOI:** 10.3389/fnmol.2017.00086

**Published:** 2017-03-29

**Authors:** Haixia Hu, Bin Ye, Le Zhang, Quan Wang, Zhiwei Liu, Suying Ji, Qiuju Liu, Jingrong Lv, Yan Ma, Ying Xu, Hao Wu, Fude Huang, Mingliang Xiang

**Affiliations:** ^1^Department of Otolaryngology and Head and Neck Surgery, Xinhua Hospital, Shanghai Jiao Tong University School of MedicineShanghai, China; ^2^Shanghai Key Laboratory of Translational Medicine on Ear and Nose DiseasesShanghai, China; ^3^Ear Institute, Shanghai Jiao Tong University School of MedicineShanghai, China; ^4^Shanghai Advanced Research Institute, University of Chinese Academy of Sciences, Chinese Academy of SciencesShanghai, China; ^5^MOE Key Laboratory of Model Animal for Disease Study, Model Animal Research Center, Nanjing UniversityNanjing, China; ^6^Institute of Neuroscience and State Key Laboratory of Neuroscience, Institute of Biological Science, Chinese Academy of SciencesShanghai, China

**Keywords:** hearing loss, *Efr3a*, spiral ganglion neuron, degeneration, neurotrophic factors

## Abstract

Sensorineural hearing loss (SNHL) is caused by an irreversible impairment of cochlear hair cells and subsequent progressive degeneration of spiral ganglion neurons (SGNs). Eighty-five requiring 3 (Efr3) is a plasma membrane protein conserved from yeast to human, and knockout of *Efr3a* was reported to facilitate the survival of hippocampal newborn neurons in adult mice. Previously, we found *Efr3a* expression in the auditory neural pathway is upregulated soon after the destruction of hair cells. Here we conducted a time-course analysis of drug-caused damage to hearing ability, hair cells and SGNs in *Efr3a* knocking down mice (*Efr3a*^−/+^, *Efr3a* KD) and their wild type littermates. Functional examination showed that both groups of mice suffered from serious hearing loss with a higher level of severity in wild type (WT) mice. Morphologic observation following drugs administration showed that both WT and *Efr3a* KD mice went through progressive loss of hair cells and SGNs, in association with degenerative changes in the perikarya, intracellular organelles, cell body conformation in SGNs, and the changes of SGNs in WT mice were more severe than in *Efr3a* KD mice. These beneficial effects of *Efr3a* KD could be ascribed to an increase in the expression of some neurotrophic factors and their receptors in *Efr3a* KD mice. Our results indicate that *Efr3a* insufficiency suppresses drug-caused SNHL neurodegeneration in association with an increase in the expression of some neurotrophic factors and their receptors, which may be targeted in the treatment of neurodegeneration.

## Introduction

The majority of the deafness patients suffer from sensorineural hearing loss (SNHL). SNHL, resulting from noise exposure, aging, genetic deficiency or ototoxic drugs, is characterized by the irreversible damage of cochlear hair cells followed by a progressive degeneration of spiral ganglion neurons (SGNs) and auditory nerve fibers (Webster and Webster, 1981; Lawner et al., 1997; Hardie and Shepherd, 1999; Fetoni et al., 2013). SGNs are the primary bipolar neurons indispensable to the pathway of auditory system that relays auditory information from the hair cells to the auditory center (Martinez-Monedero et al., 2006; Richardson et al., 2006).

In humans or other mammals, the loss of the cochlear hair cells cause permanent hearing impairment for its inability to regenerate. Cochlear implantation technology is the major option to treat hearing impairment due to the loss of cochlear hair cells, of which the efficacy depends on the number of surviving functional SGN (Nadol et al., 1989; Gantz et al., 1993; Xu et al., 2012). Therefore, the current primary strategy to further advances in cochlear implantation is to preserve SGN and induce SGN regeneration to maximize the number of functional neurons. Recently, some research progress has been made in repairing damaged SGN and regeneration of cochlear nerve fiber, such as via stem cell transplantation or exogenous neurotrophic factor (Agterberg et al., 2008; Jang et al., 2015). But there are relatively less studies about blocking or alleviating the degeneration of SGN and their synaptic terminal.

After noise, antibiotic or toxic insult to the cochlea, synapses between peripheral nerve fibers of the SGN and the hair cells in the cochlea are the first to degenerate, followed by the swollen or retraction of the cochlear nerve fiber over various time periods and finally dysfunction and loss of SGN (Dodson and Mohuiddin, 2000; Agterberg et al., 2008; Kujawa and Liberman, 2009; Nie et al., 2015). However, the molecular mechanism for the retrograde degeneration of SGN is unclear. Dying back is a common and chronic pathological process of neural degeneration which arises gradually from distal to proximal and is found in a wide variety of degenerative and toxic conditions of the peripheral and central nervous system in neurodegenerative disorders, and several possibilities have been suggested and tested to explain the dying back, involving undernourishment of the distal axon, impairment of axonal transport of organelles and vesicles, target-derived neurotrophic factors (Fischer et al., 2004; Dadon-Nachum et al., 2011). In the SNHL, retrograde degeneration of SGN subsequent to the hair cell loss in the cochlea is morphologically similar to “dying back”, but the molecular mechanism is largely unknown.

The yeast Eighty-five requiring 3 (Efr3) gene, and its *Drosophila* and mammalian homologs, *rolling blackout* (*rbo*) and *Efr3a/Efr3b* respectively, encode a membrane-localized protein that forms a protein complex with another two proteins on the plasma membrane to maintain the plasmalemmal level of phosphoinositol-4-phosphate (PI_4_P) and PI_4,5_P (Huang et al., 2004; Baird et al., 2008; Hammond et al., 2012; Nakatsu et al., 2012), which have widely direct functions in the sensory and motor nervous system, including production of IP3 and DAG, regulation of signal transduction, exocytosis, endocytosis, ion channel and neurotransmitter receptor functions, cell adhesion, and nucleation of the actin cytoskeleton (Di Paolo and De Camilli, 2006). The endocytosis mediated by PI_4,5_P is involved in a variety of processes at the cell surface (Kumari et al., 2010; McMahon and Boucrot, 2011), including axonal and neuronal degeneration, collapse, disintegration and death (Weinkove et al., 2008; Kuboyama et al., 2015). In *Drosophila*, the RBO played a pivotal role in PI_4,5_P-mediated signaling in photoreceptors, and in bulk endocytosis and macropinocytosis in neuronal and non-neuronal cells (Huang et al., 2004, 2006; Vijayakrishnan et al., 2009). In mammalian cells, Efr3 is also important in the control of G-protein-coupled receptor (GPCR)-mediated signaling by affecting the phosphorylation of GPCR (Bojjireddy et al., 2015). In adult mice, Efr3 plays an important role in the survival of newborn hippocampal neurons possibly via regulating the brain-derived neurotrophic factor (BDNF) pathway (Qian et al., 2017). Moreover, in rodents, *Efr3a* expression in the medial olivocochlear neurons in the brain stem and in the cochlear SGNs is up-regulated soon after the destruction of hair cells (Munemoto et al., 2004; Nie et al., 2015), suggesting a role of *Efr3a* in the auditory remodeling or degeneration subsequent to the deprivation of acoustic signal. In addition, we previously showed that the expression of *Efr3a* in the cochlear SGNs was increased mainly at the early stage of SGNs degeneration, which indicated that *Efr3a* may play an intermediary role in initiating cochlear SGNs degeneration (Nie et al., 2015).

In this study, we investigated the effect of partial knockout of *Efr3a* on the hair cells loss, SGN degeneration and hearing loss caused by kanamycin and furosemide treatment, and found that *Efr3a* insufficiency attenuates the progressive SGN degeneration. It is known that SGN degeneration during hearing loss could be alleviated by the treatment with neurotrophic factors, such as BDNF or neurotrophins-3 (NT-3) (Staecker et al., 1996; Richardson et al., 2005; Shepherd et al., 2005; Agterberg et al., 2008). We further examined the effect of *Efr3a* insufficiency on the expression levels of several neurotrophic factors and their receptors, and found some were elevated. Thus, our results show that *Efr3a* insufficiency attenuates drug-caused SGN degeneration possibly by up-regulating some neurotrophins-mediated signalings.

## Materials and Methods

### Generation of *Efr3a^−/+^* Mutant Mice

*Efr3a* were generated by breeding *Efr3a*^fl/fl^ mice (Qian et al., 2017) to Ella-Cre mice, which expresses efficient Cre activity in one-cell zygote stage of embryonic development (Lakso et al., 1996). The progenies with the second exon of *Efr3a* deleted and a reading frame shift in *Efr3a* mRNA (*Efr3a*^−/+^) were backcrossed to C57BL/6 mice for over 10 generations to obtain genetic background-purified *Efr3a*^−/+^ mice.

All animals were raised under standard laboratory conditions with food and water freely available. Humidity and temperature were kept constant at 60 ± 5% and 24 ± 2°C, respectively. Lights were on between 7:00 am and 7:00 pm. The care and process of animals were complied with the approval of the Institutional Authority for Laboratory Animal Care of Xinhua Hospital Affiliated Shanghai Jiao Tong University School of Medicine (Shanghai, China).

### Genotype and *Efr3a* Protein Analysis

Genotypes were determined by polymerase chain reaction (PCR) using genomic DNA from mouse tails. The mutant *Efr3a^−/+^* allele was detected using primer F 5′-TTATTTAGTATGTTGGACGA G-3′ and primer R 5′-ACAAACTTAACCTCCATGTT-3′. A 500 bp band was detected in mice with *Efr3a*^−/+^ (*Efr3a* knocking down mice, *Efr3a* KD mice), while no signal was examined in wild type mice (WT mice; data not shown).

*Efr3a* protein level was determined by fluorescent immunohistochemistry and western blotting, and the detail method referred to the previously report (Nie et al., 2015). For fluorescent immunohistochemistry, the cochleae of 8 weeks old mice without any treatment were isolated, and dealt with decalcification and dehydration. Then frozen sections (7 μm) paralleled to the modiolus were obtained. After incubation with 3% BSA, cryosections were incubated with diluted primary antibodies (1:500 mouse anti-TUJ1 (Covance) and 1:200 rabbit anti-*Efr3a* (Sigma)) at 4°C overnight and then were incubated in 1:400 FITC-conjugated donkey anti mouse and 1:400 Alexa-594 conjugated anti-rabbit goat IgG (Beyotime Biotechnology) for 1 h at room temperature. Lastly, the sections were labeled with DAPI and images were captured using a fluorescent microscope (Leica, Berlin, Germany). For western blotting, modiolus (not the intact cochlear organs) in eight cochleae were isolated, digested and separated for the further experiment.

### The Construction of SGNs Degeneration Model

After genotype analysis, all animals (8–10 weeks old) were assigned randomly into experimental or control group in both WT and *Efr3a* KD mice. The kanamycin and furosemide treatment was the same as previously described (Nie et al., 2015). Briefly, experimental animals were treated with a hypodermic injection of kanamycin (1000 mg/kg) and followed with a peritoneal injection of furosemide (400 mg/kg) 30–45 min after. All the experimental mice were divided into the 1st, 5th, 15th, 30th and 60th day groups after injection. Mice administered with same volume of saline were used as controls.

### Auditory Brainstem Response Testing

Auditory brainstem response (ABR) was carried out at the 3rd day before any injection (baseline measurement) and 5th, 15th and 60th day following the kanamycin and furosemide administration or saline injection (post measurement) in order to determine the ABR threshold shifts. It was tested using a MEB-3102 physiological response recorder (Nihon Kohden, Japan) as previously reported (Nie et al., 2015). Briefly, mice were anesthetized with ketamine (100 mg/kg) and xylazine (10 mg/kg) and kept in a sound-proof room during the test. The active electrode was inserted into the subcutaneous tissue of vertex, the reference electrode into the mastoid process, and the ground electrode into the contralateral thigh. Acoustic signal stimuli, consisting of pure tone bursts at frequencies of 4, 8, 12, 16, 24 and 32 kHz, were generated with Tucker Davis Technologies device (SigGen) with a rate of 21.1 times per second. For each test, 512 responses for every frequency were recorded and the evoked potentials were filtered with a bandpass of 100–3000 Hz. Stimuli started at 70 dB for the pretreatment groups and at 110 dB for the post-treatment groups, and decreased by 5 dB until the threshold was reached. The threshold was determined as the lowest intensity that wave III could be recorded repeatedly.

### Preparation of Cochlear Tissue

Mice cochleae were harvested at the 1st, 5th, 15th, 30th and 60th day after drugs administration. The animals were euthanized with overdose of chloral hydrate and both cochleae were dissected out. Then the cochleae were perfused and fixed in 4% phosphate buffered paraformaldehyde (pH7.5) overnight at 4°C and then decalcified in 10% ethylene diamine tetraacetic acid (EDTA) at room temperature for 1 week.

### Hair Cell Counting

The organ of Corti was carefully separated into apex turn and base turn. Immunohistochemistry for myosin VIIa were performed to evaluate cochlear hair cells. The detail procedure was carried out following the method described previously (Nie et al., 2015). The tissues were incubated in rabbit anti-myosin VIIa antibody (1:300; Proteus Bioscience) at 4°C overnight and then were incubated with Alexa-594 conjugated anti-rabbit goat IgG (1:400; Beyotime Biotechnology) for 1 h at room temperature. Afterwards, the basilar membranes were fixed on glass slides with antifade solution and images were acquired with a confocal fluorescent microscopy (Zeiss LSM710).

### SGNs Density Counting

After dissecting the bulla, the cochleae were perfused with 2.5% glutaraldehyde and fixed at least 2 h, decalcified with EDTA for 7 days and then embedded in Eponate 12. The detail protocol was performed by following the procedure described previously (Nie et al., 2015). Serial sections (1 μm thick) approximately parallel to the modiolus were obtained to count the density of SGNs. Altogether, six cross-sections from Rosenthal’s canal with an interval approximately 30 μm were taken from each cochlea. All sections were observed under a light microscope after staining with 1% toluidine blue. The SGN counts were calculated including apical, middle and basal turns. NIH Image J software was used to determine the cross-sectional area of Rosenthal’s canal. SGN density was calculated by dividing the number of perikaryon by the cross-sectional area. Five cochleae were included in each group.

### Transmission Electron Microscopy

Ultrathin (50–60 nm) sections parallel to the modiolus were prepared to study the ultrastructural morphology of SGNs. Sections were stained sequentially with uranyl acetate and lead citrate, and images were captured under a transmission electron microscope (TEM; Philips CM-120). The morphology of SGN was detected in the cochlea basal turn, a location where the influence of hair cell loss on SGN density is obvious.

Based on the close inspection of SGN from WT and KD cochleae, perikaryal area and cell circularity, two cellular characteristics of the SGN were selected for quantitative analysis. One hundred to one hundred and fifty cochlear SGNs with an obvious nucleus in three mice were counted and measured in each group. The perikaryal area and circularity were measured by NIH Image J software. The ultrastructural changes of the mitochondrial and endoplasmic reticulum morphology, lipofuscin-like granule in perikaryon were also observed. In addition, quantitative analysis of lipofuscin-like granules was performed by measuring the ratio of lipofuscin area/SGN cytoplasm area (excluding the nucleus).

### Quantitative Real Time PCR and Reverse Transcription PCR

Total RNA of the modiolus including spiral ganglia from the same brood of control WT and *Efr3a* KD mice at 8-week old was prepared using TRIzol reagent (Takara Bio Inc., Japan). Total RNA was further purified using DNase I to eliminate contaminating genomic DNA, then the cDNA was synthesized using the PrimeScript^TM^ RT Master Mix (Takara Bio Inc., Japan). A portion of the cDNAs was used as template for the real-time PCR, which were carried out with the ABI PRISM 7500 System (Thermo Fisher Scientific) using SYBR^®^ Premix Ex Taq^TM^ (Tli RNaseH Plus; Takara Bio Inc. Japan). The primer sequences of targeted genes are listed below. The relative concentration of targeted genes relative to GAPDH was determined by the 2^(−ΔΔCT)^ method. Then, another portion of the cDNAs was used for normal PCR, and the PCR products were identified with 3% agarose gel electrophoresis. The gray intensity of detected bands was quantified using Image Lab software (v 5.2; Bio-Rad, USA) and normalized them against the density of GAPDH.

• Neural growth factor (NGF):

5′ TCCACCCACCCAGTCTTCCA 3′ (forward)
5′ CCTTCCTGCTGAGCACACA 3′ (reverse)

• BDNF:

5′ GCGGCAGATAAAAAGACTGC 3′ (forward)
5′ CTTATGAATCGCCAGCCAAT 3′ (reverse)

• NT-3:

5′ CTACTACGGCAACAGAGACGCT 3′ (forward)
5′ GGTGAGGTTCTATTGGCTACCAC 3′ (reverse)

• Tyrosine kinase receptor A (TrkA):

5′ ACGGTAACAGCACATCAAGAG 3′(forward)
5′ GGAGGGCAGAAAGGAAGAG 3′(reverse)

• Tyrosine kinase receptor B (TrkB):

5′ AAGGACTTTCATCGGGAAGCTG 3′(forward)
5′ TCGCCCTCCACACAGACAC 3′(reverse)

• Tyrosine kinase receptor C (TrkC):

5′ CAACTCTCAAACACGGAGGTC 3′(forward)
5′ CCAGCATGACATCGTACACC 3′(reverse)

• GAPDH:

5′ GGTGAAGGTCGGTGTGAACG 3′ (forward)
5′ CTCGCTCCTGGAAGATGGTG 3′ (reverse)

### Western Blotting

The 8-week old WT and *Efr3a* KD mice were sacrificed and the modiolus including spiral ganglia were rapidly isolated from the cochleae. The protein was extracted as reported previously (Nie et al., 2015). Tissues were lysed in radio immunoprecipitation assay buffer (Beyotime) and homogenized, then subjected to western blotting analysis. Protein were separated by 12% SDS-PAGE gels for 120–150 min at 80 V and transferred onto polyvinylidene fluoride (PVDF) membranes (Millipore, USA). The membranes were blocked with 5% skim milk in TBST for 1 h at room temperature and incubated with primary antibodies for TrkA (Abcam, USA; 1:1000), TrkB (CST, USA; 1:1000), TrkC (CST, USA; 1:1000), NGF (Abcam, USA; 1:1000), BDNF (Sigma, USA; 1:1000), NT-3 (Santa Cruz, USA; 1:1000) and β-actin (Beyotime, 1:1000) overnight at 4°C, and then incubated with HRP-conjugated secondary antibody for 1 h at room temperature after being washed three times in TBST. The immunoreactive signals were detected using enhanced chemiluminescence system with BeyoECL Star kit (Beyotime Biotechnology) and the bands were semi-quantified using Image Lab software (v 5.2; Bio-Rad, USA).

### Statistical Analysis

The SPSS statistical software (v 19.0; SPSS Inc., Chicago, IL, USA) was used for all statistical analysis. Distributions of all data in each group were analyzed for normality using the Shapiro-Wilk test. Group comparisons between WT and KD mice at the same time point were made using Student’s *t*-test or Mann-Whitney test. Statistically significant differences of perikaryal area, circularity and lipofuscin area between experimental and control mice within WT and KD mice were determined via one-way ANOVA testing followed by Dunnett *post hoc* testing. A *p*-value of <0.05 was deemed to indicate statistical significance.

## Results

### Time Course Analysis of Auditory Brainstem Response in *Efr3a* Knockdown and Wild Type Mice Subjected to Kanamycin and Furosemide Treatment

**Figure 1:**
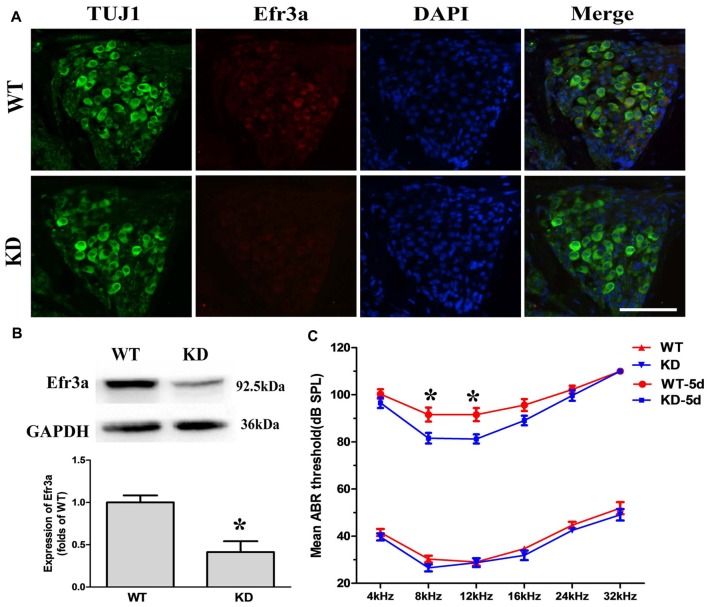
**Eighty-five requiring 3a knocking down (*Efr3a* KD) ameliorated the hearing loss induced by kanamycin and furosemide treatment. (A)** Representative fluorescent immunohistochemistry images of *Efr3a* protein in the spiral ganglion neurons (SGNs), repeated at least three times, scale bar = 50 μm; **(B)** representative western blot (top) and semi-quantification (bottom) showing the *Efr3a* expression in the spiral ganglion of cochlea, *n* = 8 for wild type (WT) and *Efr3a* KD, respectively. *T*-test, unpaired, two tail; **(C)** mean auditory brainstem response (ABR) thresholds to sound stimulations at different frequencies. KD and WT represent *Efr3a* KD and WT mice without drug treatment respectively, while KD-5d and WT-5d represent *Efr3a* KD and WT mice with drugs treatment at the 5th day posterior to drugs application respectively. *n* = 16 for each data point, Mann-Whitney test or unpaired *T*-test, following normality test; Data are represented by mean ± SEM, “*” indicates *p* < 0.05 between *Efr3a* KD and WT mice at the same frequency.

Unconditional *Efr3a* knockout mice (*Efr3a*^−/−^) are embryonic lethal. Thus, we used *Efr3a*^−/+^ heterozygotes, here named as *Efr3a* KD mice, for the following experiments. We first examined the expression level of *Efr3a* protein in *Efr3a* KD mice and their WT littermates. Immunofluorescence staining in the frozen sections showed that, compared with WT mice, the expression of *Efr3a* was obviously decreased in the *Efr3a* KD mice (Figure 1A). Consistently, quantitative analysis of *Efr3a* protein in cochlea by western blotting showed an intense band from the cochleae of WT mice, but a weaker band from the cochleae of KD mice at 92.5 kDa position, the expression of *Efr3a* was decreased by approximately 50% (*p* < 0.05) in the *Efr3a* KD mice, confirming the reduction of *Efr3a* in *Efr3a* KD mice (Figure 1B).

**Figure 2:**
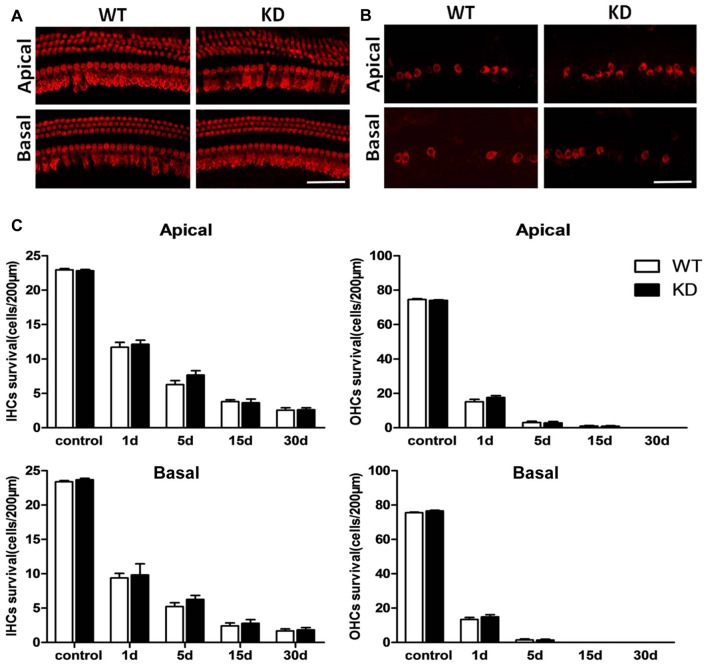
***Efr3a* reduction did not change the loss of hair cells caused by drugs treatment** Representative myosin VIIa immuno-labeled hair cells in the cochleae of the WT (WT) and *Efr3a* KD (KD) mice without drug treatment **(A)** and with drugs treatment at the 5th day **(B)**, scale bar = 50 μm. **(C)** Quantification of VIIa immuno-labeled hair cells in non-treated (control) and drug-treated WT and KD mice at the 1st, 5th, 15th and 30th day posterior to drugs application, *n* ≥ 5 for each data point. Data are represented by mean ± SEM. No significant difference in density of hair cells in both apical and basal turns of cochlea between *Efr3a* KD and WT mice were observed.

Then, a time course analysis of the effect of *Efr3a* KD on the hearing impairment induced by kanamycin and furosemide treatment through measuring the ABR threshold was conducted. Total 16 *Efr3a* KD mice and 16 WT were used. Before drugs treatment, the hearing function observed in both WT and *Efr3a* KD mice are similar to that previously described for C57BL/6J (Park et al., 2010; Mistry et al., 2014; Xiong et al., 2014), the ABR thresholds at pure tones of 4, 8, 12, 16, 24 and 32 kHz frequencies between the two groups were indistinguishable (Figure 1C, bottom), indicating partial deletion of *Efr3a* did not significantly change the function of the auditory system. Five days after drugs treatment, the ABR thresholds in both groups were markedly increased, demonstrating a severe hearing loss in both groups. Interestingly, the ABR thresholds in KD mice were relatively lower than those in WT mice, especially at 8 kHz (91.56 ± 2.95 dB vs. 81.56 ± 2.27 dB, *t* test, *p* = 0.012) and 12 kHz (91.56 ± 2.80 dB vs. 81.25 ± 1.96 dB, *t* test, *p* = 0.005; Figure 1C, top). Fifteen and 60 days after drugs treatment, however, the ABR thresholds at all six frequencies in almost all animals were higher than 85 dB, and no significant difference of ABR thresholds between WT and KD mice was observed, which might be due to the loss of vast majority of hair cells in both strains of mice (Figure 2). Nevertheless, the data demonstrate that *Efr3a* reduction temporarily ameliorated the hearing impairment caused by kanamycin and furosemide.

### *Efr3a* Reduction did Not Change the Loss of Hair Cells Caused by Drugs Treatment

We conducted immunofluorescence staining in whole mounts of the Corti organ with an antibody against Myosin VIIa to label hair cells in both *Efr3a* KD and WT mice with and without drug treatment. As shown in Figures 2A,C, the density, morphology and arrangement of hair cells in both groups without drug treatment were indistinguishable, indicating *Efr3a* KD did not affect the development of hair cells. We also did a time course analysis of the density of hair cells in both groups of mice with drugs treatment at the 1st, 5th, 15th and 30th day after drugs administration. Starting from the 1st day, a progressive severe loss of hair cells occurred in both groups (Figures 2B,C). The density of inner hair cells (IHCs) in both apical and basal cochlea was reduced by about 50% at the 1st day, followed by about 50% decrease in the density of residual hair cells by the 5th day, and a further 50% reduction by the 15th day, then no further obvious loss of hair cells. The destruction of outer hair cells (OHCs) was faster and severer than the IHCs, more than 70% by the 1st day, and almost complete loss by the 15th day. At each time point posterior to drugs treatment, there was no significant change in density of residual inner or OHCs in both apical and basal turns of cochlea between *Efr3a* KD and WT mice (Figure 2C).

### *Efr3a* Reduction Suppressed SGN Degeneration Following Hair Cell Loss

**Figure 3:**
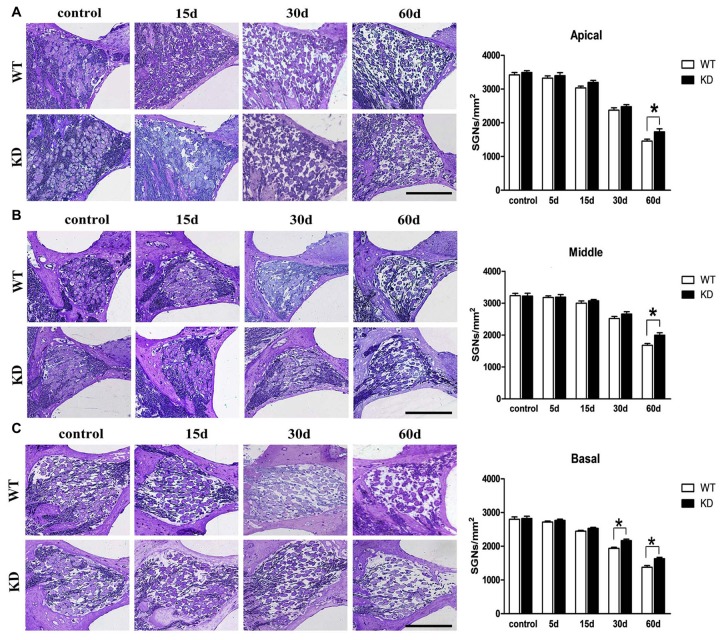
***Efr3a* reduction attenuated the loss of SGNs caused by drugs treatment.** Serial light microscopy changes of the SGNs from WT mice vs. *Efr3a* KD mice from apical **(A)**, middle **(B)** and basal **(C)** regions of the cochlea, scale bar = 100 μm. The quantification of the SGN densities in each region is shown at the right. SGN density showed a progressive decrease in both strains, but earlier and more severe in WT mice **(A–C)**. Data are represented by mean ± SEM, *n* = 5 for each data point, *T*-test, unpaired, two tail; “*” indicates *p* < 0.05.

Hair cells are innervated by the SGNs, which are known to degenerate over time following the loss of hair cells. To test whether *Efr3a* reduction could produce a protective role against the SGN degeneration, we conducted toluidine blue staining and light imaging, then quantitative analysis of the densities of SGNs in Rosenthal’s canal at the 5th, 15th, 30th and 60th day following drugs administration. We counted SGNs in the apical, middle and basal regions of Rosenthal’s canal. At the 5th day posterior to the drugs administration, the densities of SGN in both WT and *Efr3a* KD mice did not change significantly, but progressively decreased to much lower values at the following time points, 45% in WT and 53% in KD group in the apical turn at the 60th day (*t* test, *p* = 0.026; Figure 3A); 51% in WT and 61% in KD group in the middle turn at the 60th day (*t* test, *p* = 0.008; Figure 3B); 69% in WT and 76% the in KD group in the basal turn at the 30th day (*t* test, *p* = 0.002) and 49% in WT and 57% in KD group in the basal turn at the 60th day (*t* test, *p* = 0.026; Figure 3C).

**Figure 4:**
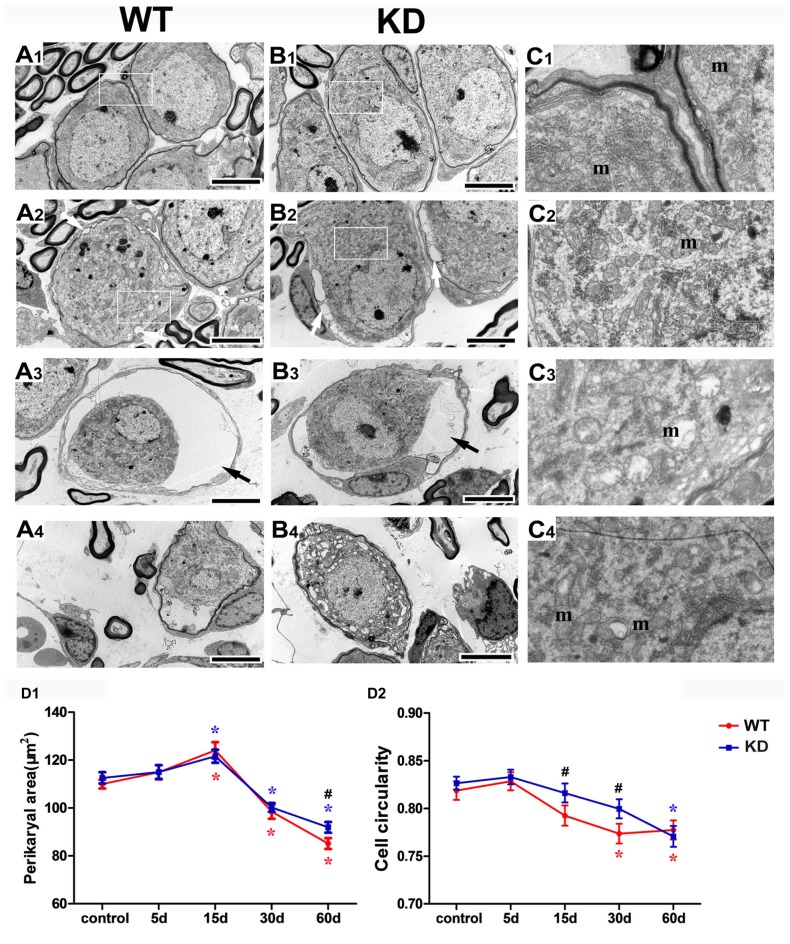
***Efr3a* reduction ameliorated the structural alterations of SGNs following hair cell loss.** Representative transmission electron microscope (TEM) images of the SGNs in the cochlea from WT and *Efr3a* KD (KD) mice without **(A1,B1)** and with drugs treatment at the 15th **(A2,B2)**, 30th **(A3,B3)** and 60th **(A4,B4)** day posterior to drugs application. **(C1–C4)** is a close view of the boxed areas in **(A1,B1,A2,B2)** respectively. White arrows indicate the cytoplasmic vacuoles gathered at the edge of the cytoplasm of the SGNs, with a few swelling and scattered mitochondria (m), while black arrows indicate the balloon-like appearance around the perikarya of SGNs. Scale bar = 5 μm in **(A1–A4)** and **(B1–B4)**. Quantitative time-course analysis of the perikaryal area **(D1)** and cell circularity **(D2)**. Data are represented by mean ± SEM, *n* ≥ 100 SGNs for each data point, unpaired *T*-test (two tail) or Mann-Whitney test between WT and KD group at the same time point, and one-way ANOVA testing followed by Dunnett *post hoc* testing between experimental and control group within WT and KD mice. Blue “*” indicates *p* < 0.05 compared with control in *Efr3a* KD mice, red “*” indicates *p* < 0.05 compared with control in WT mice, and black “^#^” indicates *p* < 0.05 between *Efr3a* KD and WT mice in each time point. WT vs. *Efr3a* KD: *p* = 0.038 at the 60th day for perikaryal area, *p* = 0.026 at the 15th day and *p* = 0.018 at the 30th day for cell circularity.

We further studied the SGN degeneration with TEM. The TEM images of SGNs were taken from the cochlear basal turn, where the effect of hair cell loss on SGN density is most prominent. In WT and *Efr3a* KD mice without drug treatment, several SGNs lie typically close to each other, intracellular organelles, such as mitochondria, are intact, chromatin is distributed evenly in the prominent nucleus and the perikaryon is surrounded by the myelin sheath of the peripheral process of a Schwann cell (Figures 4A1,B1,C1,C2). However, drugs treatment induced a series of time-dependent intracellular changes in the SGNs in both WT and *Efr3a* KD mice. At the 5th day, similar to our previous report (Nie et al., 2015), the perikarya of only few neurons were slightly or moderately enlarged (data not shown) in both WT and *Efr3a* KD mice. At the 15th day, the perikarya further enlarged, abnormal mitochondria with rarefaction of the matrix and partial loss of cristae as well as cytoplasm vacuolization became evident, lipofuscin accumulated, and the cell body of SGNs deformed (reflected by the change in cell circularity) in both WT mice and *Efr3a* KD mice, but more severe in WT mice (Figures 4A2,B2,C3,C4,D1,D2, 5A,B). At 30th day and 60th day, the perikarya shrinked gradually, and other degenerative changes became more and more severe in both WT mice and *Efr3a* KD mice, and largely more severe in WT mice (Figures 4A3,B3,A4,B4,D1,D2, 5A,B). These data demonstrate that *Efr3a* reduction reduced the drug-induced degeneration in SGNs.

**Figure 5:**
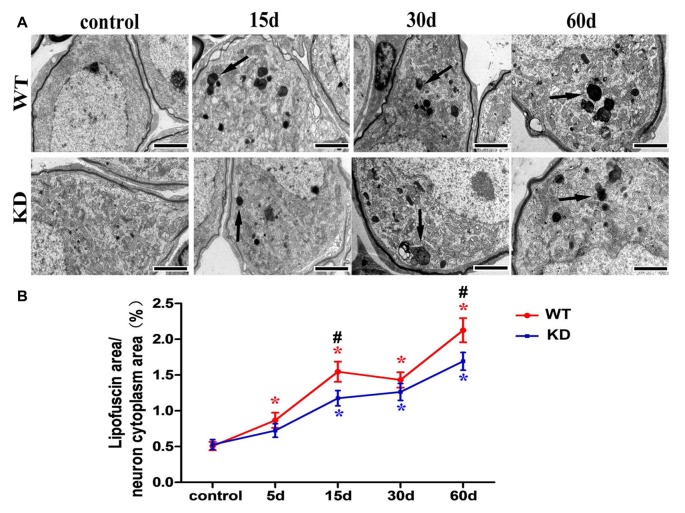
***Efr3a* reduction suppressed the formation of lipofuscin in SGNs following hair cell loss. (A)** Representative TEM images showing lipofuscin-like granule (black arrow) in the perikarya of SGNs. Scale bars = 2 μm. **(B)** Quantitative time-course analysis of the ratio of lipofuscin area/SGN cytoplasm area (excluding the nucleus). Data were represented by mean ± SEM, *n* ≥ 50 SGNs for each data point, unpaired *T*-test (two tail) between WT and KD group at the same time point, and one-way ANOVA testing followed by Dunnett *post hoc* testing between experimental and control group within WT and KD mice. Blue “*” indicates *p* < 0.05 compared with control in *Efr3a* KD mice, red “*” indicates *p* < 0.05 compared with control in WT mice, and black “^#^” indicates *p* < 0.05 between *Efr3a* KD and WT mice in each time point. WT vs. *Efr3a* KD: *p* = 0.043 at the 15th day and *p* = 0.045 at the 60th day.

### *Efr3a* Reduction Increased the Expression of Some Neurotrophic Factors and their Receptors

**Figure 6:**
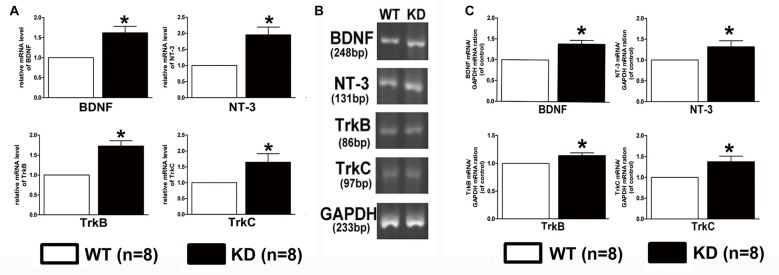
***Efr3a* reduction upregulated the expression of some neurotrophins and their receptors at mRNA levels.** Quantification of the mRNA levels of brain-derived neurotrophic factor (BDNF), neurotrophins-3 (NT-3), tyrosine kinase receptor B (TrkB) and tyrosine kinase receptor C (TrkC) in WT and *Efr3a* KD mice by real-time polymerase chain reaction (PCR; **A**) and reverse transcription (RT)-PCR **(B,C)**, the representative images of the electrophoresis of RT-PCR products are shown in **(B)**. Data are represented by mean ± SEM, *n* = 8, *T*-test, unpaired, two tail. “*” indicates *p* < 0.05 between *Efr3a* KD and WT mice.

**Figure 7:**
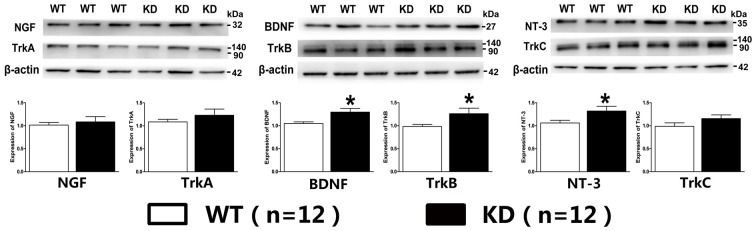
***Efr3a* reduction upregulated the expression of some neurotrophins and their receptors at protein levels.** The representative immunoreactive bands and quantification of the protein levels of neural growth factor (NGF), BDNF, NT-3, tyrosine kinase receptor A (TrkA), TrkB and TrkC in WT and *Efr3a* KD mice by western blotting. Data are represented by mean ± SEM, *n* = 12, *T*-test, unpaired, two tail. “*” indicates *p* < 0.05 between *Efr3a* KD and WT mice.

Lack of target-derived neurotrophic factors has been suggested and tested to explain the dying back process of neurons. Given the report that the expression of BDNF and its receptor TrkB was found to be increased in the brain of mice with nestin-CRE mediated complete knockout of *Efr3a* (Qian et al., 2017), Quantitative real-time PCR was performed to examine the mRNA of NGF, BDNF, NT-3 and their receptors, TrkA, TrkB and TrkC, in the spiral ganglions from the same brood of control WT and *Efr3a* KD mice without drug treatment. Indeed, the mRNA levels of BDNF and NT-3 and their receptors, but not NGF and TrkA, were significantly elevated in *Efr3a* KD mice (Figure 6A). Then we re-confirmed the changes by reverse transcription PCR (RT-PCR) and electrophoresis, and found similar elevation in BDNF, NT-3, TrkB and TrkC (Figures 6B,C). Further more, the protein levels of BDNF, NT-3 and TrkB, but not NGF, TrkA and TrkC, in *Efr3a* KD mice were higher than those in WT mice (Figure 7).

## Discussion

Aminoglycoside antibiotics have substantial ototoxic effects. A single dose injection of aminoglycoside immediately following by injection of a loop diuretic induces hair cell loss rapidly, and subsequent progressive degeneration of SGNs and their auditory nerve fibers in chinchilla, rats, guinea pigs and mice at relatively slower speed, which have been frequently used to model the disease of SNHL for studying disease mechanism and therapeutic efficacy (McFadden et al., 2004; Glueckert et al., 2008; Layman et al., 2015; Wang et al., 2015). In the present study, we, with the co-administration of a single dose of kanamycin and furosemide, successfully and rapidly destroyed vast majority of cochlear hair cells by 15 days, consequently induced a progressive degeneration of SGNs in mice with a similar time course as reported in the previous studies. At the 5th day after drugs administration, WT animals developed a severe hearing impairment, at 15th day the ABR thresholds at all frequencies were higher than 85 dB, and remained high until the 60th day. In parallel to the hearing loss, there was a progressive loss of IHCs: about 50% IHCs disappeared by the 1st day, followed by 50% reduction of the residual IHCs by the 5th day, a further 50% reduction by the 15th day, then not much change during the 15th–30th days. The loss of OHCs was relatively faster and more severe, over 70% loss by the 1st day, and almost complete loss by the 15th day. Consequently, starting from the 5th day, the SGNs degenerated over time, and the ultrastructure of SGNs underwent stereotypic degenerative changes in the perikarya, intracellular organelles, cell body conformation.

*Efr3a* KD temporarily suppressed the rapid loss of hearing ability, and the progressive and relatively slower degenerative alterations in SGNs in the model of hearing loss, indicating *Efr3a* KD produced a beneficial effect against the neurodegeneration of SGNs induced by aminoglycoside antibiotics. Moreover, *Efr3a* KD significantly increased the expression of BDNF, NT-3 and their receptors, TrkB and TrkC at both mRNA and protein levels, but not the expression of NGF and TrkA, which is consistent with a recent study that complete knockout of *Efr3a* in brain neurons elevated the expression levels of BDNF and TrkB, and suppressed the apoptosis of hippocampal newborn neurons in adult mice (Qian et al., 2017).

Neurotrophic factors and their tyrosine kinase receptors play essential roles in the establishment of neuron number through their control of cell survival/death during neuronal development and degeneration (Huang and Reichardt, 2001; Schimmang et al., 2003; Ito and Enomoto, 2016). The degeneration of SGN in SNHL was considered to be a consequence of absence of neurotrophic factor from the hair cells, which is supported by many other studies showing that the ultrastructural changes and loss of SGNs in SNHL could be attenuated by the application of BDNF or NT-3 (Staecker et al., 1996; Richardson et al., 2005; Shepherd et al., 2005; Agterberg et al., 2008; Landry et al., 2011; Fukui et al., 2012; van Loon et al., 2013). In addition, previous studies showed that *Efr3a* expression was up-regulated in the lateral superior olive within 2 days after the removal of cochleae (Munemoto et al., 2004) and in SGNs within 5 days after the co-administration of kanamycin and furosemide (Nie et al., 2015). Therefore, we speculate that *Efr3a* may negatively regulate the expression of NT-3, BDNF, TrkB and TrkC, and the beneficial effect of *Efr3a* KD might be ascribed to an elevation of the corresponding downstream signalings.

It is well known that Efr3/RBO forms a plasma membrane localized complex with PI4KIIIα and a scaffold protein to maintain the levels of plasmalemmal PI_4_P and PI_4,5_P, particularly PI_4_P (Baird et al., 2008; Hammond et al., 2012; Nakatsu et al., 2012). Whether and how this protein complex regulates the expression of neurotrophins and their receptors, and in turn regulates the down-stream signalings by controlling plasmalemmal levels of PI_4_P and PI_4,5_P remain to be determined.

Except at a narrow window of stimulating frequencies and at the 5th day after drugs treatment, there was a close correlation between ABR thresholds and the densities of SGNs and hair cells in WT and *Efr3a* KD mice at all stimulating frequencies and at all the days thereafter. The explanation could be that at the 5th day, the functional state of SGNs and hair cells with higher levels of neurotrophins in *Efr3a* KD mice was better than that in WT mice. *Efr3a* KD produced moderate beneficial effect on the survival of SNGs, but not on that of hair cells. The explanation is that the detrimental effect of ototoxic drugs at high dose on the preservation of hair cell density was too strong be suppressed by the beneficial effect of *Efr3a* insufficiency due to the higher susceptibility of hair cells to the drugs than SGNs and other cells in the body. Nevertheless, the functional state of hair cells with higher levels of neurotrophins in *Efr3a* KD mice might be still better than that in WT mice at the 5th days after drugs injection. Therefore, under sound stimulation at a narrow range of frequencies at the 5th day after drugs application, the ABR thresholds in *Efr3a* KD mice were significantly lower than those in WT mice.

Taken together, our study indicated that decrease of *Efr3a* attenuates the degeneration of SGN after hair cell loss, possibly by up-regulating the expression of some neurotrophic factors and their receptors.

## Author Contribution

MX and FH conceived the project; FH wrote the manuscript; MX, FH, HH and BY designed the experiments; HW provided technical and platform supports. HH participated in manuscript preparation and did most of the experiments along with BY except ABR measurement and hair cell counting, which was carried out by JL and YM, respectively; HH raised the animals along with QW; LZ, SJ and ZL generated the *Efr3a*^−/+^ mice and purified the genetic background under the instruction of YX. All authors listed, have made substantial, direct and intellectual contribution to the work, and approved it for publication.

## Conflict of Interest Statement

The authors declare that the research was conducted in the absence of any commercial or financial relationships that could be construed as a potential conflict of interest.

## Abbreviations

ABR: auditory brainstem response
BDNF: brain-derived neurotrophic factor
EDTA: Ethylene Diamine Tetraacetic Acid
*Efr3*: Eighty-five requiring 3
*Efr3a* KD: *Efr3a* knocking down
IHCs: inner hair cells
NGF: neural growth factor
NT-3: neurotrophins-3
OHCs: outer hair cells
PCR: polymerase chain reaction
PI_4_P: phosphoinositol-4-phosphate
RBO: rolling blackout
RT-PCR: reverse transcription PCR
SGN: spiral ganglion neuron
SNHL: sensorineural hearing loss
TEM: transmission electron microscope
TrkA: tyrosine kinase receptor A
TrkB: tyrosine kinase receptor B
TrkC: tyrosine kinase receptor C
WT mice: wild type mice.
